# Efficacy of rt-PA versus Urokinase in early time windows for minimally invasive intracerebral hemorrhage evacuation and exploratory TAFI pharmacogenomics

**DOI:** 10.3389/fneur.2026.1830505

**Published:** 2026-08-03

**Authors:** Yonggang Jiao, Huanwen Chen, Dongpeng Qiu, Shengming Zhang, Jian Gao

**Affiliations:** 1 Department of Neurology, The Affiliated Guangdong Second Provincial General Hospital of Jinan University, Guangzhou, China; 2 Department of Neurology, Yangshan Hospital of the Affiliated Guangdong Second Provincial General Hospital of Jinan University, Yangshan, China; 3 Department of Health Management, The Affiliated Guangdong Second Provincial General Hospital of Jinan University, Guangzhou, China

**Keywords:** intracerebral hemorrhage, minimally invasive surgery, pharmacogenomics, recombinant tissue plasminogen activator, thrombin-activatable fibrinolysis inhibitor

## Abstract

**Background:**

Minimally invasive hematoma evacuation with local thrombolysis is increasingly used for spontaneous intracerebral hemorrhage (ICH), yet comparative data on thrombolytic agents across early time windows remain limited. Whether the thrombin-activatable fibrinolysis inhibitor (TAFI) Thr325Ile polymorphism modulates thrombolytic efficacy in this setting is unknown.

**Methods:**

We conducted a retrospective comparative cohort study involving 120 patients with supratentorial ICH. Following minimally invasive hematoma aspiration, patients were treated with either rt-PA (*n* = 60) or urokinase (*n* = 60). Each group was stratified by onset-to-surgery time into ultra-acute (3 to 6 h), acute (6 to 24 h), and subacute (24 to 72 h) subgroups. Neurological and functional outcomes, hematoma clearance, perihematomal edema, and TAFI genotypes were analyzed. Pre-specified exploratory subgroup analyses by dominant hemisphere and by hematoma volume stratum (30–50 mL vs. 51–80 mL) were also performed. Effect sizes with 95% confidence intervals (CIs) are reported alongside *p-*values, and the Bonferroni method was applied to control the family-wise error rate for primary between-group comparisons.

**Results:**

rt-PA was associated with better neurological and functional outcomes than urokinase from discharge onward across all three time windows (all Bonferroni-adjusted *p* < 0.017), with higher hematoma clearance and lower perihematomal edema. The difference in 3-month NIHSS score within the ultra-acute subgroup was 5.24 points (95% CI: 4.30–6.18; Cohen’s *d* = 4.06). Ultra-acute treatment produced the most favorable outcomes regardless of agent. The TAFI Ile325 (TT) genotype was enriched in the ultra-acute rt-PA subgroup (80.00% vs. 22.22% acute and 29.63% subacute; *p* = 0.009; odds ratio for TT carriage vs. urokinase in the ultra-acute subgroup = 10.00 [95% CI: 1.80–55.71]) and positively correlated with hematoma clearance in that window (*r* = 0.670, *p* = 0.006). No genotype–outcome association was observed with urokinase.

**Conclusion:**

rt-PA was associated with more favorable outcomes than urokinase for minimally invasive ICH evacuation in this retrospective cohort, with the ultra-acute window conferring the greatest benefit. The TAFI Ile325 genotype may represent a candidate pharmacogenomic marker for enhanced rt-PA response during this early period. These hypothesis-generating findings require validation in prospective, multicenter studies.

## Introduction

Spontaneous intracerebral hemorrhage (ICH) is a severe stroke subtype caused by non-traumatic rupture of brain vessels, leading to hematoma formation within the parenchyma (1). It represents the second most common type of stroke after ischemic stroke and carries high mortality and disability rates (2). The 2021 Global Burden of Disease report estimated 3.444 million new ICH cases worldwide, with an age-standardized mortality rate of 39.1 per 100,000, imposing a substantial societal and economic burden (3).

For supratentorial ICH, timely hematoma evacuation is important to alleviate mass effect and potentially improve outcomes (4). However, the role of traditional craniotomy remains controversial. The STICH II trial found no benefit for early surgical removal of superficial lobar hemorrhages without intraventricular extension (5, 6). Consequently, attention has shifted toward less invasive techniques. Minimally invasive puncture, guided by CT, followed by local administration of thrombolytic agents to liquefy and drain the clot, has emerged as a promising strategy with the potential to reduce injury to surrounding brain tissue (7, 8). The MISTIE III trial demonstrated that image-guided catheter drainage with alteplase achieved meaningful hematoma reduction and was safe, although the primary functional outcome was not improved at the cohort level (9). Subsequent analyses and the recent ENRICH trial of minimally invasive parafascicular surgery have further refined patient-selection criteria (10). The Chinese Guidelines for the Diagnosis and Treatment of Cerebral Hemorrhage (2019) and the contemporary 2022 AHA/ASA Guideline for the Management of Spontaneous Intracerebral Hemorrhage (11) both acknowledge minimally invasive approaches as reasonable in selected patients (12).

Despite growing interest in minimally invasive thrombolysis, key questions remain. In particular, there is a lack of comparative data evaluating different thrombolytic regimens across specific early time windows, namely the ultra-acute (3–6 h), acute (6–24 h), and subacute (24–72 h) phases after ICH onset. In Chinese clinical practice, urokinase is widely used for this purpose given its local availability, lower cost (13), and extensive clinical experience, although it lacks broad international recognition for ICH thrombolysis. In contrast, recombinant tissue plasminogen activator (rt-PA) is the globally established thrombolytic for acute ischemic stroke and is increasingly being investigated for ICH hematoma evacuation (14). A direct comparison of these two agents within the context of minimally invasive ICH surgery is therefore warranted.

Furthermore, individual variability in treatment response, potentially influenced by genetic factors, remains poorly understood. Thrombin-activatable fibrinolysis inhibitor (TAFI) is an endogenous inhibitor of rt-PA–mediated fibrinolysis (15). A common polymorphism in the TAFI gene (Thr325Ile) results in variant enzymes with differing thermal stability and antifibrinolytic activity, which could plausibly modulate the efficacy of rt-PA in dissolving intracerebral hematomas (16). Because hematoma composition evolves over time, the interaction between rt-PA efficacy and an individual’s fibrinolytic profile may vary across treatment windows. To date, no study has examined whether TAFI genotypes are associated with rt-PA–mediated hematoma clearance at specific time points after ICH.

Therefore, we conducted this retrospective comparative study with a dual aim: (1) to compare the hematoma-clearing efficacy and clinical outcomes of rt-PA versus urokinase when administered via minimally invasive puncture across three defined time windows (ultra-acute, acute, subacute), and (2) to explore, in a hypothesis-generating fashion, whether the TAFI Thr325Ile polymorphism is associated with the therapeutic effect of rt-PA within these early phases.

## Materials and methods

### Study design and setting

This was a single-center, retrospective comparative cohort study conducted at the Yangshan Branch of Guangdong Provincial Second People’s Hospital (Guangdong, China). Medical records of patients who underwent minimally invasive hematoma aspiration surgery between April 1, 2023, and March 31, 2025, were systematically reviewed.

The choice of thrombolytic agent, rt-PA or urokinase, was determined by the attending neurosurgeon based on clinical judgment and was not randomized. In routine practice at our center, the decision reflected a combination of four factors: (1) drug availability at the time of admission, as rt-PA stocks were intermittently constrained during the earlier portion of the study period owing to supply variations; (2) the treating neurosurgeon’s prior experience and familiarity with each agent; (3) financial considerations communicated by the patient or family, as urokinase carries a substantially lower acquisition cost in our region and is not always fully covered by insurance; and (4) family preference following standardized counseling regarding the two available options, their evidence base, and their respective costs. No formal allocation rule was applied.

This research was approved by the Ethics Committee of the Yangshan Branch of the Second People’s Hospital of Guangdong Province. The study was conducted in accordance with the principles outlined in the Declaration of Helsinki (2013 revision). Each patient, or an authorized family member, where the patient lacked decision-making capacity, provided written informed consent prior to enrollment. Separate written consent was obtained for genetic testing of the TAFI Thr325Ile polymorphism. All patient data were anonymized during analysis to ensure confidentiality.

### Participants

A total of 120 patients who underwent hematoma aspiration surgery during the study period and met the eligibility criteria were included. Participants were selected from consecutive admissions, as detailed below. In addition to the variables described in the outcome sections, data on hemisphere laterality of the hemorrhage (left vs. right), hematoma location (lobar vs. deep), and baseline hematoma volume stratum (30–50 mL vs. 51–80 mL) were extracted from the medical records for use in exploratory subgroup analyses.

#### Inclusion criteria

Patients were included if they met all of the following criteria:

1) Met the minimally invasive treatment standards for cerebral hemorrhage as stipulated in the Chinese Guidelines for the Diagnosis and Treatment of Cerebral Hemorrhage (2019 Edition) (12).
2) Age ≥ 18 years and ≤ 80 years.
3) Not pregnant at the time of enrollment.
4) Diagnosed with spontaneous supratentorial intracerebral hemorrhage (ICH), with hematoma volume ranging from 30 mL to 80 mL as measured on initial computed tomography (CT).
5) Glasgow Coma Scale (GCS) score (17) ≥ 5 points and National Institutes of Health Stroke Scale (NIHSS) score (18) ≥ 6 points at presentation.
6) Preoperative computed tomography angiography (CTA) did not detect aneurysms or arteriovenous malformations in the vascular territory responsible for the hemorrhage.

#### Exclusion criteria

Patients were excluded if any of the following applied:

1) Secondary ICH caused by trauma, aneurysms, arteriovenous malformations, or cerebral venous thrombosis.
2) Subtentorial (infratentorial) bleeding.
3) ICH score (19) ≥ 3 points, indicative of very poor prognosis.
4) Abnormal coagulation parameters or clinically evident bleeding tendency.
5) Severe intraventricular hemorrhage (IVH) requiring external ventricular drainage.
6) Pre-existing severe neurological deficits, defined as a modified Rankin Scale (mRS) score (20) ≥ 2 points prior to the index ICH event.
7) Severe cardiac, hepatic, renal, or pulmonary dysfunction rendering the patient unable to tolerate surgery.
8) Advanced-stage malignancy or other life-threatening illness with an estimated survival of 1 year or less.

### Study grouping and time-window stratification

**Figure 1:**
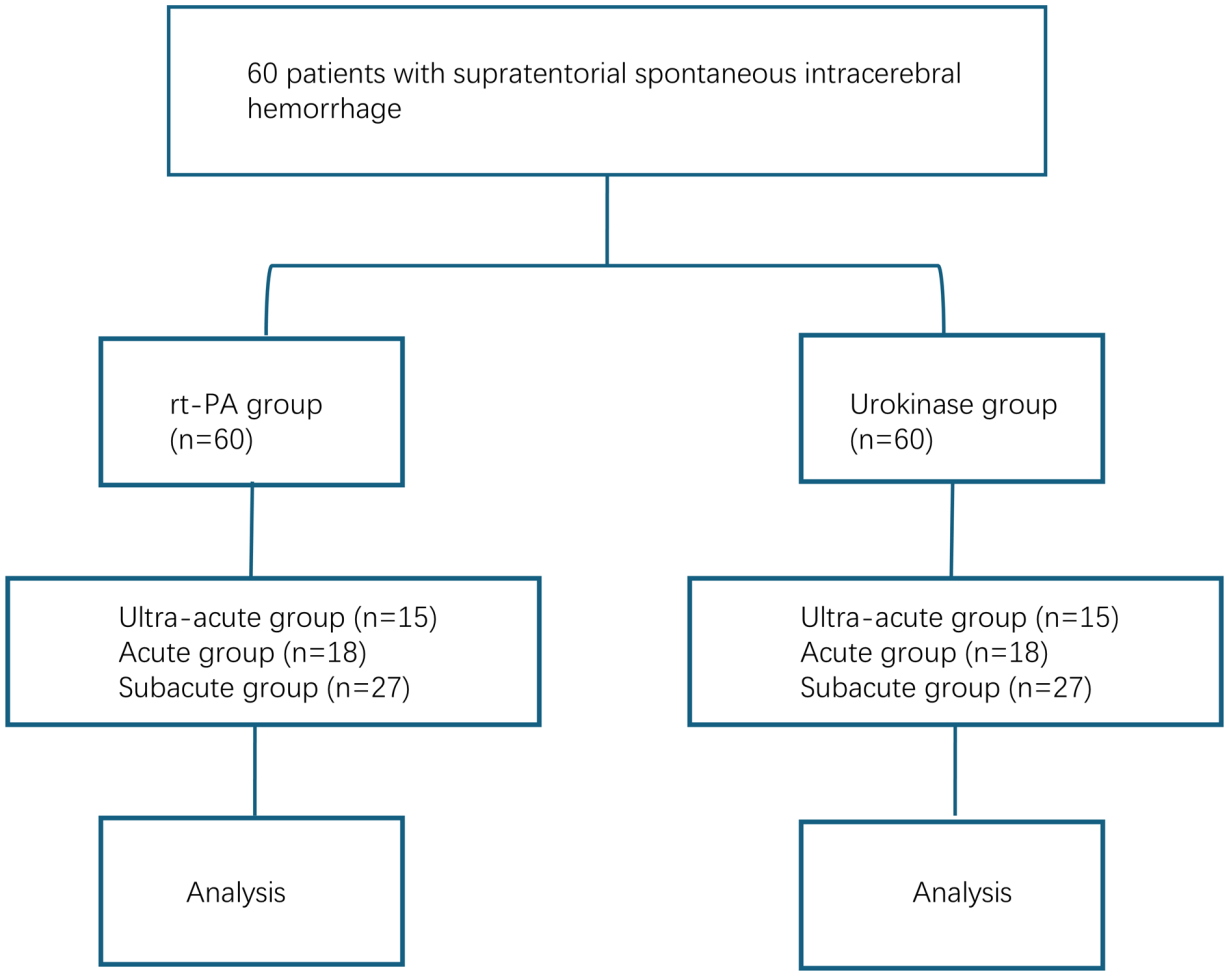
Study flow diagram. Schematic representation of patient enrollment, group allocation, and time-window subgroup stratification. A total of 120 patients with spontaneous supratentorial intracerebral hemorrhage (ICH) undergoing minimally invasive hematoma aspiration were divided into the rt-PA group (*n* = 60) and the urokinase group (*n* = 60). Each group was further stratified by onset-to-surgery time into ultra-acute (3–6 h), acute (>6–24 h), and subacute (>24–72 h) phase subgroups.

Patients were categorized into two groups based on the thrombolytic agent received during minimally invasive puncture: the rt-PA group (*n* = 60) and the urokinase group (*n* = 60). Each group was further stratified into three subgroups according to the onset-to-surgery time: the ultra-acute phase subgroup (3 h ≤ onset-to-surgery time ≤ 6 h), the acute phase subgroup (6 h < onset-to-surgery time ≤ 24 h), and the subacute phase subgroup (24 h < onset-to-surgery time ≤ 72 h). The terms “ultra-acute,” “acute,” and “subacute” are used consistently throughout the manuscript according to these definitions. The study flow and subgroup distribution are presented in Figure 1. Onset time was defined as the time of witnessed symptom onset or, for unwitnessed events, the last known time the patient was neurologically intact.

### Blinding and bias minimization

To minimize ascertainment bias, outcome assessors were blinded to both the treatment group assignment and the TAFI genotyping results throughout the study period. Genotype data were maintained by an independent statistician and were not disclosed to the clinical team or outcome assessors until the final database was locked for analysis. Although the retrospective design precluded blinding of the treating surgeons, all radiological assessments of hematoma volume and perihematomal edema were performed by an experienced neuroradiologist who was blinded to the patient’s group allocation and clinical outcomes.

### Sample size considerations

As this was a retrospective study, all consecutive patients meeting the eligibility criteria during the two-year study period were included. No *a priori* sample size calculation was performed; the final sample size of 120 patients was determined by the number of eligible patients treated at our center during the defined study period. The resulting subgroup sizes (ranging from *n* = 14 to *n* = 29 per treatment-by-time-window stratum) were acknowledged as modest; accordingly, subgroup analyses were designated as exploratory and hypothesis-generating.

### Intervention protocols

#### Minimally invasive puncture procedure

The optimal puncture plane, target point, and trajectory were determined using preoperative CT guidance. Following routine antiseptic preparation, sterile draping, and local anesthesia, a YL-1 type intracranial hematoma puncture needle of appropriate length (Beijing Wanteifu Technology Co., Ltd., Beijing, China) was selected. The needle was advanced through the skull along the predetermined trajectory using an electric drill. Upon reaching the target, a plastic needle core was inserted, and the liquefied component of the hematoma was carefully aspirated layer by layer according to the pre-calculated depth. Postoperative cranial CT was performed immediately to confirm the position of the puncture needle and evaluate residual hematoma.

#### rt-PA administration protocol

Recombinant tissue plasminogen activator was administered through the indwelling catheter at a dose of 1 mg diluted in 1 mL of normal saline every 8 h, with a maximum cumulative dose of 9.0 mg over a treatment duration not exceeding 72 h. After each instillation, the drainage tube was clamped for 2 h to allow contact between the thrombolytic agent and the residual clot, after which the drain was reopened. The decision to continue or discontinue rt-PA administration was guided by the results of serial CT re-examinations. The puncture needle was removed when the residual hematoma volume decreased to ≤ 15 mL or when no residual hematoma was visualized surrounding the needle on follow-up imaging.

#### Urokinase administration protocol

Urokinase was administered through the indwelling catheter at a dose of 20,000 IU diluted in 2 mL of normal saline every 8 h, with a maximum cumulative dose of 180,000 IU over a treatment duration not exceeding 72 h. The clamping, drainage, and discontinuation protocols were identical to those described for the rt-PA group.

#### Postoperative management

Highly consistent postoperative care measures were implemented for both groups in strict accordance with the Chinese Guidelines for the Diagnosis and Treatment of Cerebral Hemorrhage (2019 Edition) (7), aiming to minimize treatment-related confounding. Standard postoperative management included blood pressure control, blood glucose management, and administration of tranexamic acid for hemostasis. Antihypertensive agents were titrated to maintain systolic blood pressure below 140 mmHg in accordance with guideline recommendations. If follow-up CT imaging indicated rebleeding, thrombolytic treatment was immediately discontinued.

### Blood sample collection and DNA extraction

Three milliliters of fasting venous blood was collected from each study participant on the day of enrollment (within 24 h of admission), anticoagulated with ethylenediaminetetraacetic acid dipotassium salt (EDTA-K2), and stored at −80 °C until analysis. Genomic DNA was extracted from whole blood specimens according to the manufacturer’s instructions provided with the DNA extraction kit. The purity and concentration of the extracted DNA were assessed using a NanoVue ultra-micro spectrophotometer (GE Healthcare, UK). Samples with an A260/A280 ratio greater than 1.8 were deemed of sufficient purity for downstream analysis. Qualified DNA specimens were aliquoted and stored at −80 °C until genotyping.

### TAFI gene polymorphism detection

The Thr325Ile polymorphism of the TAFI gene was genotyped using polymerase chain reaction–restriction fragment length polymorphism (PCR-RFLP). Primers were synthesized by Sigma (USA) based on sequences retrieved from the PrimerBank database. The forward primer sequence was 5′-GGGTCTTCGAGGCACCTTTG-3′ and the reverse primer sequence was 5′-AACTGCCGGAGTTTGATGGG-3′. The *β*-actin reference gene was amplified using the forward primer 5′-CCTGGCACCCAGCACAAT-3′ and the reverse primer 5′-GCCGATCCACACGGAGTACT-3′.

Each PCR reaction was carried out in a total volume of 25 μL, consisting of 0.5 μL template DNA, 2.0 μL dNTP mixture (2.5 mmol/L; Takara, Japan), 2.5 μL 10 × Ex Taq buffer (without Mg^2+^), 1.5 μL MgCl₂ (25 mmol/L), 0.25 μL TaKaRa Ex Taq DNA polymerase (2.5 units), 1.0 μL each of forward and reverse primers, and 17.25 μL nuclease-free water. Thermocycling conditions comprised initial denaturation at 94 °C for 2 min, followed by 35 cycles of denaturation at 94 °C for 30 s, annealing at 52 °C for 30 s, and extension at 72 °C for 5 min. A final extension step was performed at 72 °C for 10 min. Amplified products were stored at 4 °C until restriction digestion.

For RFLP analysis, the PCR amplification products were digested with SpeI restriction endonuclease at 37 °C for 3 h. The digestion patterns were as follows: the homozygous Ile325 (TT) variant yielded a single fragment of 453 bp; the homozygous Thr325 (CC) variant yielded two fragments of 236 bp and 217 bp; and the heterozygous Thr325Ile (CT) variant yielded three fragments of 453 bp, 236 bp, and 217 bp. Fragments were resolved by 2% agarose gel electrophoresis and visualized by ethidium bromide staining under ultraviolet illumination. A random subset of 10% of samples was re-genotyped to confirm reproducibility, yielding 100% concordance.

### Radiological assessment of hematoma and perihematomal edema

Cranial CT scanning was performed using the auditory–eyebrow line (orbitomeatal line) as the baseline, with a slice thickness of 5.0 mm. Volumetric quantification of the hematoma and surrounding edema was performed using the semi-automated volume analysis module on the IntelliSpace Portal Philips Nebula workstation (Philips Healthcare, Best, The Netherlands) by an experienced neuroradiologist blinded to treatment allocation and clinical outcomes.

On each consecutive axial CT slice, two separate regions of interest (ROIs) were manually delineated. The hyperdense hematoma core was outlined based on its distinct high attenuation [typically >45 Hounsfield Units (HU)]. A second, larger ROI was drawn to encompass the entire area of abnormal hypodensity surrounding the hematoma, representing the combined region of hematoma and perihematomal edema, with the outer boundary defined by the visible margin of hypodensity (typically <30 HU) relative to the normal brain parenchyma. The software automatically calculated the area (cm^2^) for each delineated region on every slice, and total volumes were computed by summation of the products of the area and slice thickness (5 mm). The following derived variables were calculated: volume of perihematomal edema = volume of the combined (hematoma + edema) region − volume of the hematoma; relative edema volume = volume of edema / preoperative hematoma volume; and hematoma clearance rate = (preoperative hematoma volume − residual hematoma volume)/preoperative hematoma volume × 100%.

To quantify interobserver reliability of the volumetric measurements, a randomly selected subset of 30 scans (25% of the total cohort) was independently re-measured by a second experienced neuroradiologist who was blinded to the first reader’s values, to treatment allocation, and to clinical outcomes. Agreement between readers was quantified using the two-way random-effects, single-rater, absolute-agreement intraclass correlation coefficient (ICC(2,1)) with 95% CIs. Interobserver agreement was excellent for both hematoma volume (ICC = 0.94; 95% CI: 0.88–0.97) and perihematomal edema volume (ICC = 0.91; 95% CI: 0.82–0.95). The mean absolute between-reader difference was 0.7 mL for hematoma volume and 1.1 mL for edema volume (see Supplementary Table S3). These results support the reliability of the volumetric analyses presented below.

### Outcome measures

#### Primary outcomes

Primary outcomes encompassed neurological function, daily living abilities, and overall prognosis. Neurological function was assessed using the NIHSS score (continuous), GCS score (ordinal), and mRS score (ordinal) at five time points: before surgery, 24 h postoperatively, 1 week postoperatively, at hospital discharge, and 3 months postoperatively. Daily living abilities were evaluated at 3 and 6 months postoperatively using the Modified Barthel Index (MBI) (21), scored on a 100-point scale with higher scores denoting greater functional independence. Prognosis was assessed at 3 and 6 months postoperatively using the Glasgow Outcome Scale (GOS) (22), scored from 1 to 5 points, with scores of 4 and 5 classified as favorable outcomes. The proportion and distribution of the three TAFI gene subtypes (Thr325 [CC], Thr325Ile [CT], and Ile325 [TT]) in each subgroup were also considered a primary endpoint.

#### Secondary outcomes

Secondary outcomes included hematoma clearance rate, volume of perihematomal edema, and relative edema volume, each assessed on the final postoperative CT prior to needle removal. Additionally, the correlation between the three TAFI gene subtypes and hematoma clearance rate in the three time-window subgroups of the rt-PA group was analyzed as a secondary, exploratory endpoint.

### Statistical analysis

All analyses were performed using SPSS version 17.0 (IBM Corp., Armonk, NY, USA). Continuous variables were assessed for normality using the Shapiro–Wilk test (for subgroups with *n* < 50) and for homogeneity of variance using the Levene test. Normally distributed continuous data were expressed as mean ± standard deviation (SD) and compared between two independent groups using the independent-samples *t*-test, or among three or more groups using one-way analysis of variance (ANOVA) with *post-hoc* Bonferroni correction for pairwise comparisons.

Ordinal outcome variables (mRS, GCS, and GOS scores) were analyzed using the Mann–Whitney *U* test for two-group comparisons and the Kruskal–Wallis test for three-group comparisons, with Dunn’s *post-hoc* test applied where indicated. Summary values for ordinal scales are presented as mean ± SD purely as descriptive measures to maintain comparability with the existing intracerebral hemorrhage literature, which conventionally reports these scores in this format; however, all inferential testing for these variables relied on non-parametric methods, and the non-parametric test results constitute the primary basis for statistical inference. Categorical data were expressed as frequencies and percentages, and between-group comparisons were conducted using the *χ*^2^ test or Fisher’s exact test as appropriate.

Effect sizes were calculated alongside *p-*values for the primary outcome comparisons. Cohen’s d (with 0.2, 0.5, and 0.8 interpreted as small, medium, and large effects, respectively) was computed for between-group differences in continuous variables; mean differences with 95% CIs are reported for the principal between-group contrasts. For categorical and genotype comparisons, odds ratios (ORs) with 95% CIs were calculated using the Woolf–Haldane approximation, with continuity correction applied to sparse cells when required. Pearson correlation coefficients (*r*) with 95% CIs were used for correlation analyses.

To control the family-wise error rate for the principal pre-specified comparison of rt-PA versus urokinase across the three time windows, the Bonferroni method was applied to the three primary between-group contrasts (Bonferroni-adjusted significance threshold *α* = 0.05/3 = 0.017). Subgroup analyses, including the time-window, TAFI genotype, dominant-hemisphere, and hematoma-volume-stratum analyses, were pre-specified as exploratory and hypothesis-generating. Given the limited statistical power within these strata and the risk of attenuating real signals through overly conservative adjustment, formal correction for multiple comparisons was not applied to these exploratory analyses; results are reported with unadjusted *p-*values.

Pearson correlation analysis was conducted to examine the relationship between the three TAFI gene subtypes and hematoma clearance rate within the time-window subgroups of the rt-PA group. To account for potential confounding, analysis of covariance (ANCOVA) was performed for key continuous outcomes (hematoma clearance rate, NIHSS scores at follow-up), with treatment group and/or time window as fixed factors and baseline hematoma volume and location (lobar vs. deep) as covariates. Hardy–Weinberg equilibrium (HWE) was assessed for the TAFI genotype distribution in each subgroup using the *χ*^2^ goodness-of-fit test. Two additional exploratory subgroup analyses were performed to assess the robustness of the principal between-group comparison: (1) stratification by dominant-hemisphere involvement (left vs. right hemisphere, with the left hemisphere designated as dominant for the predominantly right-handed cohort) and (2) stratification by baseline hematoma volume (30–50 mL vs. 51–80 mL). The 51-mL threshold was chosen based on established prognostic literature underpinning the volume component of the ICH Score (23, 24). Results are presented in Supplementary Tables S1, S2. A two-tailed *p* < 0.05 (or, where applicable, the Bonferroni-adjusted threshold) was considered statistically significant.

## Results

### Baseline characteristics

A total of 120 patients met the eligibility criteria and were included in the final analysis, with 60 patients in the rt-PA group and 60 in the urokinase group. As presented in Table 1, the two treatment groups were well balanced with respect to demographic and clinical characteristics at baseline. No statistically significant differences were observed between the rt-PA and urokinase groups in terms of sex distribution, age, prevalence of hypertension, diabetes mellitus, prior stroke, coronary heart disease, smoking history, alcohol consumption, systolic and diastolic blood pressure upon admission, blood glucose level upon admission, hemisphere laterality, hematoma location (lobar vs. deep), or hematoma volume stratum (all *p* > 0.05).

**Table 1:** Baseline data of patients between the rt-PA group and urokinase group.

Items	rt-PA group (*n* = 60)	Urokinase group (*n* = 60)	*χ*^2^/*t*	*p*
Gender			0.009	0.921
Male	33 (55.00)	32 (53.33)		
Female	27 (45.00)	28 (46.67)		
Age (years)	62.65 ± 5.64	62.89 ± 5.72	0.232	0.817
History of hypertension	42 (70.00)	40 (66.67)	0.154	0.694
History of diabetes	30 (50.00)	34 (56.67)	0.535	0.464
History of stroke	29 (48.33)	34 (56.67)	0.835	0.360
History of coronary heart disease	43 (71.67)	37 (61.67)	1.350	0.245
History of smoking	40 (66.67)	34 (56.67)	1.269	0.259
History of drinking	32 (53.33)	38 (63.33)	1.234	0.266
SBP upon admission (mmHg)	153.28 ± 28.86	153.24 ± 28.78	0.007	0.993
DBP upon admission (mmHg)	79.58 ± 8.76	79.32 ± 8.58	0.164	0.869
Blood glucose upon admission (mmol/L)	6.18 ± 2.03	6.23 ± 2.16	0.130	0.896
Hemisphere laterality			0.137	0.711
Left (dominant)	35 (58.33)	33 (55.00)		
Right	25 (41.67)	27 (45.00)		
Hematoma location			0.140	0.708
Lobar	22 (36.67)	24 (40.00)		
Deep	38 (63.33)	36 (60.00)		
Hematoma volume stratum			0.137	0.711
30–50 mL	35 (58.33)	33 (55.00)		
51–80 mL	25 (41.67)	27 (45.00)		

Values are presented as *n* (%) or mean ± SD. Hemisphere laterality was classified by side of hemorrhage; the left hemisphere was designated as dominant for the predominantly right-handed cohort. Hematoma location was classified as lobar (cortical/subcortical) versus deep (basal ganglia, thalamus, periventricular). SBP, systolic blood pressure; DBP, diastolic blood pressure.

Similarly, within each treatment group, the three time-window subgroups (ultra-acute, acute, and subacute) demonstrated comparable baseline characteristics (Table 2). In the rt-PA group, the ultra-acute, acute, and subacute subgroups comprised 15, 18, and 27 patients, respectively. In the urokinase group, the corresponding subgroups comprised 14, 17, and 29 patients, respectively. No significant inter-subgroup differences were identified for any of the recorded baseline variables (all *p* > 0.05), supporting the comparability of the strata for subsequent outcome analyses.

**Table 2:** Baseline data of patients in the three subgroups of the rt-PA and urokinase group.

*rt-PA group* (*n* = 60)					
Item	Ultra-acute (*n* = 15)	Acute (*n* = 18)	Subacute (*n* = 27)	*χ*^2^/*F*	*p*
Male	8 (53.33)	10 (55.56)	15 (55.56)	0.022	0.988
Female	7 (46.67)	8 (44.44)	12 (44.44)		
Age (years)	62.53 ± 5.56	62.62 ± 5.63	62.73 ± 5.69	0.005	0.995
History of hypertension	10 (66.67)	12 (66.67)	20 (74.07)	0.388	0.823
History of diabetes	8 (53.33)	10 (55.56)	12 (44.44)	0.622	0.732
History of stroke	7 (46.67)	9 (50.00)	13 (48.15)	0.037	0.981
History of coronary heart disease	11 (73.33)	13 (72.22)	19 (70.37)	0.045	0.977
History of smoking	9 (60.00)	11 (61.11)	20 (74.07)	1.216	0.544
History of drinking	8 (53.33)	9 (50.00)	15 (55.56)	0.133	0.935
SBP upon admission (mmHg)	153.15 ± 28.83	153.23 ± 28.96	153.31 ± 29.08	0.000	0.999
DBP upon admission (mmHg)	79.26 ± 8.65	79.38 ± 8.72	80.06 ± 8.81	0.053	0.948
Blood glucose upon admission (mmol/L)	6.23 ± 2.27	6.06 ± 2.09	6.16 ± 1.98	0.028	0.972
Hemisphere—Left (dominant)	9 (60.00)	10 (55.56)	16 (59.26)	0.078	0.962
Hematoma location—Lobar	5 (33.33)	7 (38.89)	10 (37.04)	0.119	0.942
Hematoma volume—30–50 mL	9 (60.00)	10 (55.56)	16 (59.26)	0.078	0.962

*Urokinase group* (*n* = 60)					
Item	Ultra-acute (*n* = 14)	Acute (*n* = 17)	Subacute (*n* = 29)	*χ*^2^/*F*	*p*
Male	7 (50.00)	10 (58.82)	15 (51.72)	0.298	0.861
Female	7 (50.00)	7 (41.18)	14 (48.28)		
Age (years)	62.35 ± 5.42	63.08 ± 5.59	61.08 ± 5.41	0.812	0.449
History of hypertension	9 (64.29)	11 (64.71)	20 (68.97)	0.134	0.935
History of diabetes	7 (50.00)	9 (52.94)	18 (62.07)	0.324	0.850
History of stroke	8 (57.14)	9 (52.94)	17 (58.62)	0.378	0.827
History of coronary heart disease	8 (57.14)	11 (64.71)	18 (62.07)	0.189	0.909
History of smoking	7 (50.00)	10 (58.82)	17 (58.62)	0.330	0.847
History of drinking	8 (57.14)	11 (64.71)	19 (65.52)	0.304	0.858
SBP upon admission (mmHg)	153.21 ± 28.96	153.36 ± 29.15	153.28 ± 28.72	0.000	0.999
DBP upon admission (mmHg)	78.95 ± 8.43	79.04 ± 8.67	79.19 ± 8.57	0.004	0.995
Blood glucose upon admission (mmol/L)	6.19 ± 2.21	6.27 ± 2.32	6.22 ± 2.18	0.005	0.994
Hemisphere—Left (dominant)	8 (57.14)	9 (52.94)	16 (55.17)	0.062	0.970
Hematoma location—Lobar	5 (35.71)	7 (41.18)	12 (41.38)	0.146	0.930
Hematoma volume—30–50 mL	8 (57.14)	9 (52.94)	16 (55.17)	0.062	0.970

Values are presented as *n* (%) or mean ± SD. SBP, systolic blood pressure; DBP, diastolic blood pressure.

Interobserver reliability for the volumetric CT measurements was excellent, with ICC(2,1) values of 0.94 (95% CI: 0.88–0.97) for hematoma volume and 0.91 (95% CI: 0.82–0.95) for perihematomal edema volume across the 30 scans re-measured by a second blinded reader (Supplementary Table S3).

### Neurological function outcomes

#### Within-group comparisons across time-window subgroups

**Figure 2:**
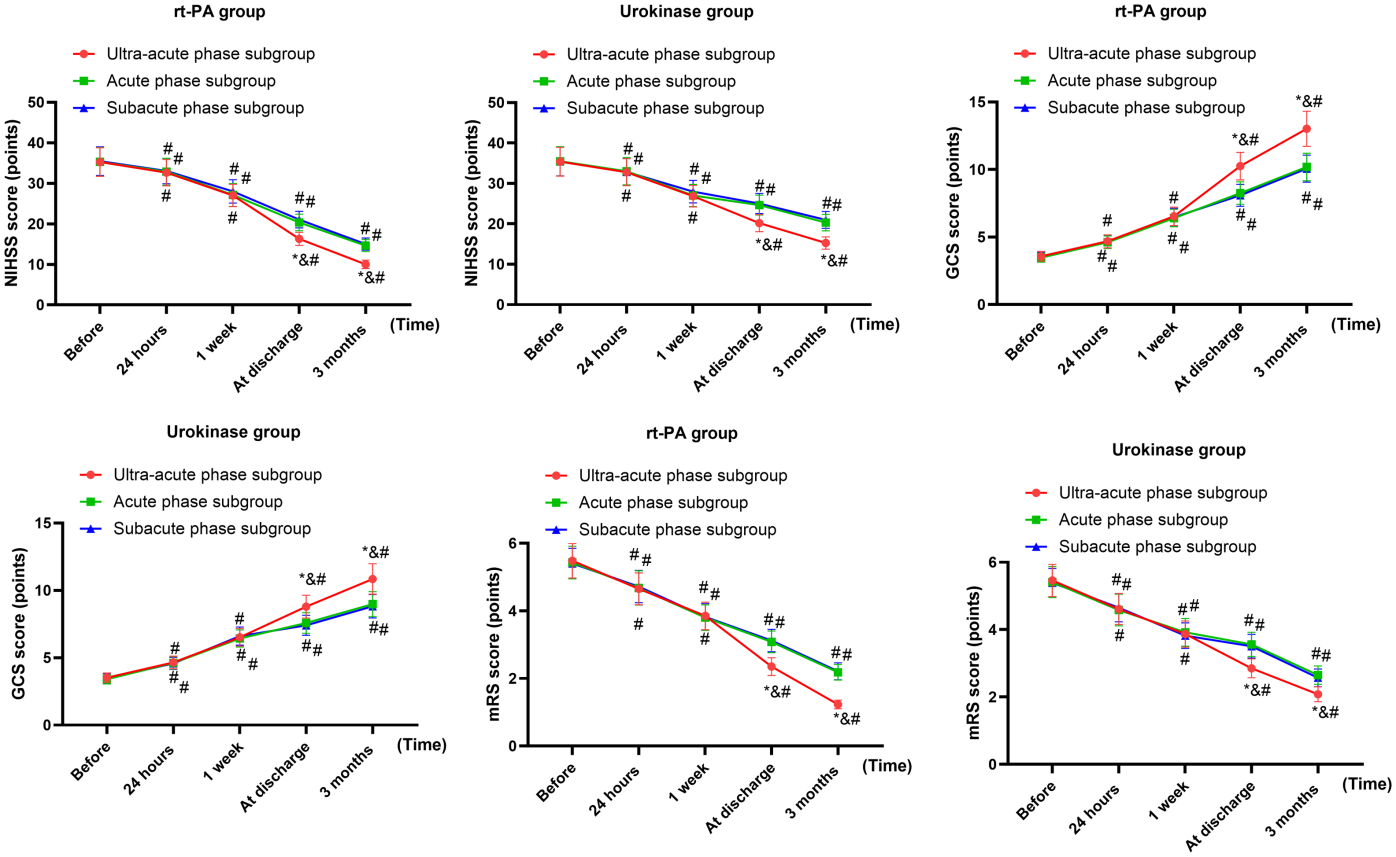
Longitudinal neurological function scores across time-window subgroups within each treatment group. NIHSS, mRS, and GCS scores assessed before surgery, at 24 h, 1 week, discharge, and 3 months postoperatively. Sample sizes for the rt-PA group were as follows: Ultra-acute *n* = 15, acute *n* = 18, subacute *n* = 27. Sample sizes for the urokinase group were as follows: Ultra-acute *n* = 14, acute *n* = 17, subacute *n* = 29. ^#^*p* < 0.05 vs. before surgery; *p < 0.05 vs. acute subgroup; ^&^*p* < 0.05 vs. subacute subgroup. One-way ANOVA with Bonferroni *post-hoc* was used for NIHSS; Kruskal–Wallis with Dunn’s *post-hoc* was used for mRS and GCS.

Neurological function was assessed longitudinally using the NIHSS, mRS, and GCS at five time points (Figure 2). At baseline (before surgery), no significant differences in NIHSS, mRS, or GCS scores were detected among the ultra-acute, acute, and subacute subgroups within either the rt-PA or the urokinase group (all *p* > 0.05), confirming comparable severity of neurological impairment at presentation.

All three subgroups in both treatment arms demonstrated statistically significant improvements from baseline at each postoperative assessment. The NIHSS and mRS scores declined progressively, while GCS scores increased steadily from 24 h postoperatively through to the 3-month follow-up (all *p* < 0.05 vs. baseline). Importantly, at discharge and at 3 months postoperatively, a significant time-window effect emerged: patients treated within the ultra-acute phase exhibited lower NIHSS scores (rt-PA: 16.32 ± 1.62 at discharge, 10.02 ± 1.03 at 3 months; urokinase: 20.18 ± 2.12 at discharge, 15.26 ± 1.52 at 3 months), lower mRS scores, and higher GCS scores compared with those treated in the acute or subacute phases (*p* < 0.05), as illustrated in Figure 2. No statistically significant differences were observed between the acute and subacute subgroups at any time point.

#### Between-group comparisons (rt-PA vs. Urokinase)

**Figure 3:**
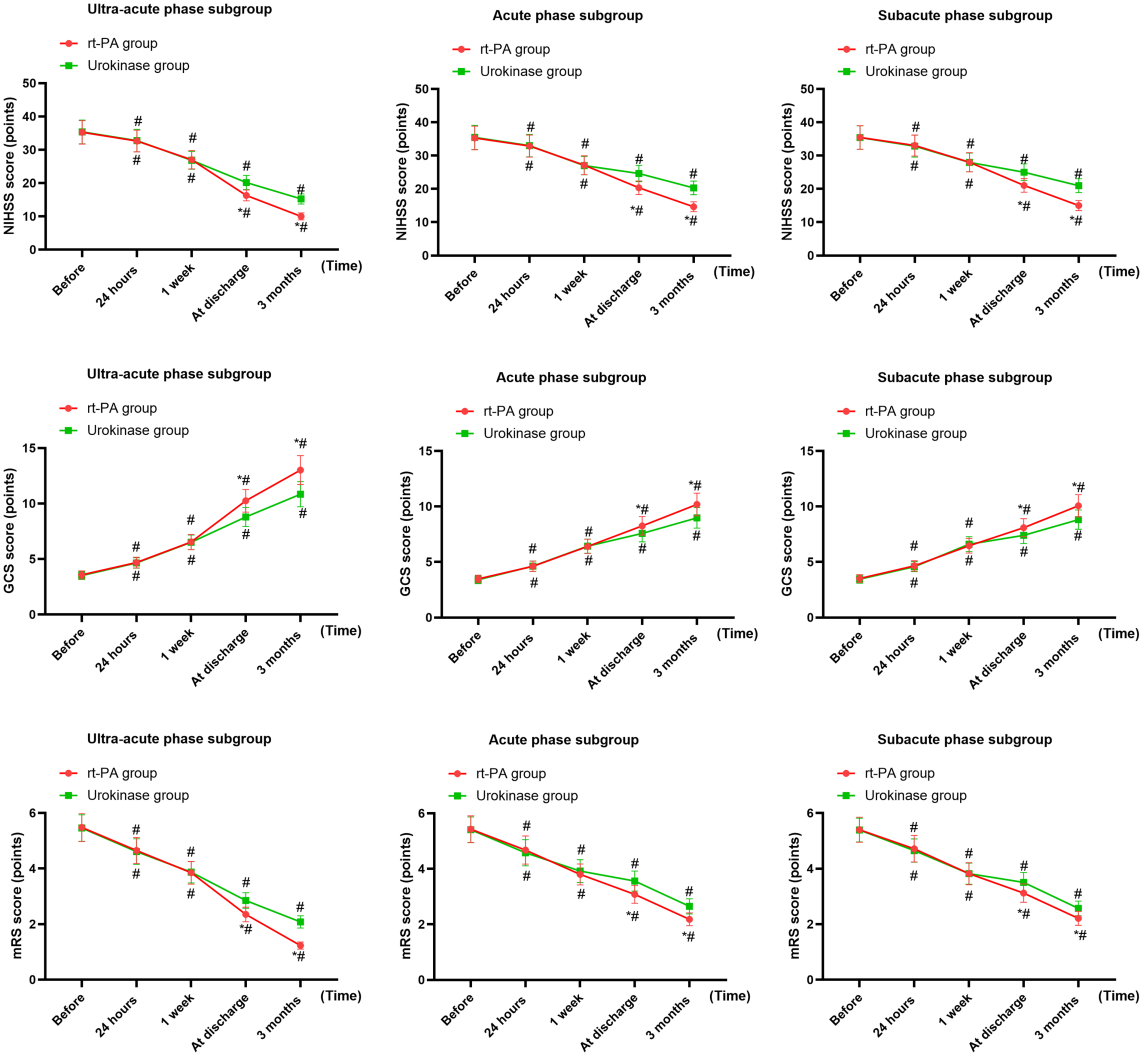
rt-PA versus urokinase comparison of neurological function scores within each time-window subgroup. Same neurological scales and assessment time points as in Figure 2. ^#^*p* < 0.05 vs. before surgery; *p < 0.05 vs. urokinase group (Bonferroni-adjusted threshold *α* = 0.017 applied to the three primary between-group contrasts). Independent-samples *t*-test was used for NIHSS; Mann–Whitney *U* test was used for mRS and GCS.

Direct comparison of the two treatment arms within each time-window subgroup revealed no significant differences in NIHSS, mRS, or GCS scores at baseline, 24 h, or 1 week postoperatively (all *p* > 0.05), indicating comparable early neurological trajectories (Figure 3). However, from the point of discharge onward, the rt-PA group consistently outperformed the urokinase group across all three time-window subgroups, and these differences remained significant after Bonferroni adjustment (all adjusted *p* < 0.017). In the ultra-acute phase subgroup, the rt-PA group had significantly lower NIHSS scores at discharge (16.32 ± 1.62 vs. 20.18 ± 2.12; mean difference 3.86 points, 95% CI: 2.49–5.23; Cohen’s *d* = 2.05) and at 3 months (10.02 ± 1.03 vs. 15.26 ± 1.52; mean difference 5.24 points, 95% CI: 4.30–6.18; Cohen’s *d* = 4.06). A corresponding pattern was observed in the acute phase subgroup (discharge NIHSS: 20.36 ± 2.05 vs. 24.65 ± 2.46; 3-month NIHSS: 14.65 ± 1.48 vs. 20.32 ± 2.04) and the subacute phase subgroup (discharge NIHSS: 21.06 ± 2.06 vs. 25.04 ± 2.48; 3-month NIHSS: 15.02 ± 1.51 vs. 20.98 ± 2.08), with all comparisons remaining significant after Bonferroni adjustment.

Parallel trends were observed for the mRS and GCS scores. At discharge and at 3 months postoperatively, the rt-PA group demonstrated significantly lower mRS scores and higher GCS scores than the urokinase group across all three subgroups (Figure 3). These findings indicate that rt-PA was associated with better neurological recovery than urokinase, irrespective of the treatment time window.

### Daily living abilities and prognosis

#### Within-group comparisons across time-window subgroups

**Figure 4:**
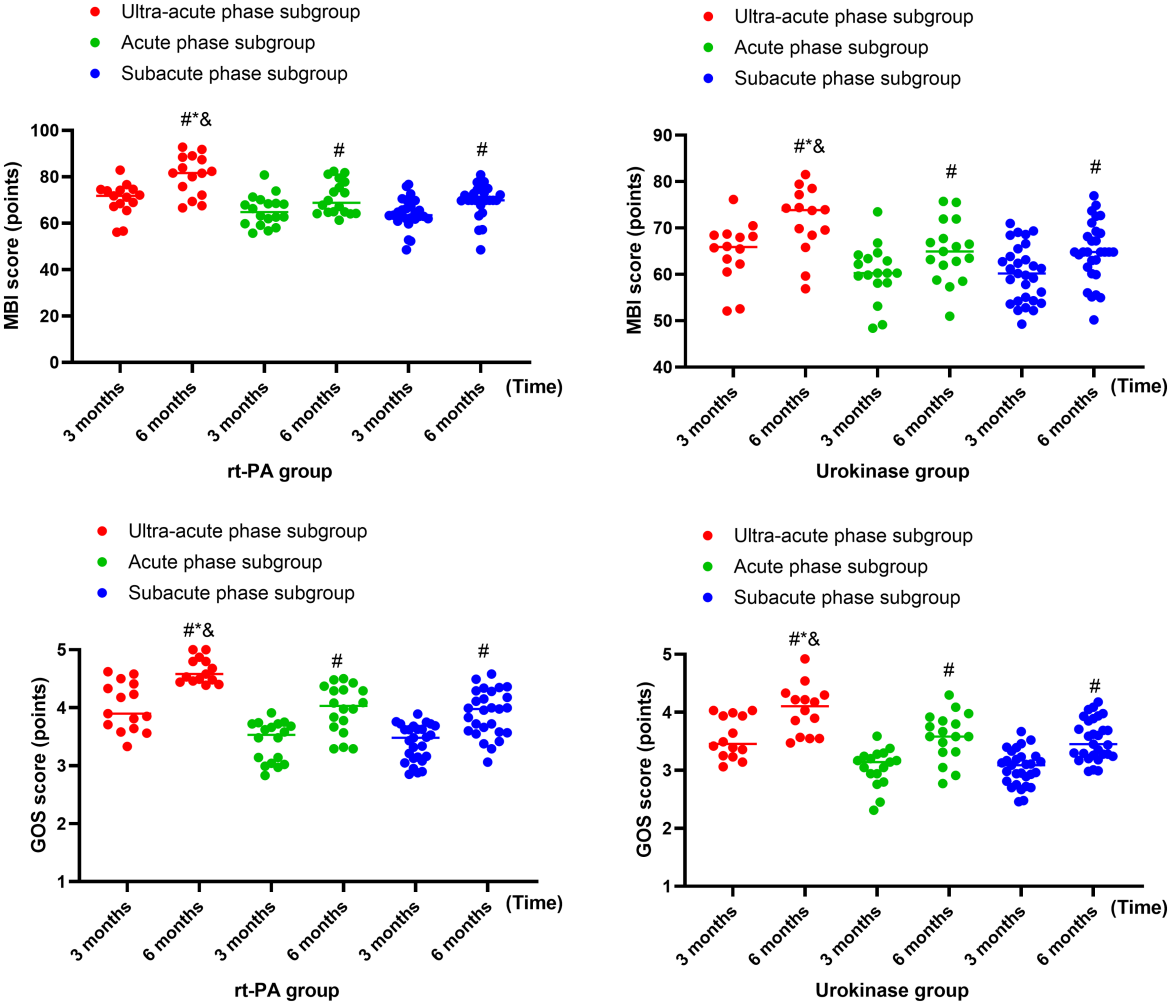
Functional outcomes (MBI and GOS) across time-window subgroups within each treatment group. MBI and GOS scores at 3 and 6 months postoperatively. Sample sizes as in Figure 2. ^#^*p* < 0.05 vs. 3 months; *p < 0.05 vs. acute subgroup; ^&^*p* < 0.05 vs. subacute subgroup. One-way ANOVA with Bonferroni *post-hoc* was used for MBI; Kruskal–Wallis with Dunn’s *post-hoc* was used for GOS.

Functional outcomes at medium-term follow-up were evaluated using the MBI and GOS at 3 and 6 months postoperatively (Figure 4). In both treatment groups, the MBI and GOS scores improved significantly from 3 months to 6 months after surgery (all *p* < 0.05), reflecting continued functional recovery over the follow-up period. The ultra-acute phase subgroup consistently achieved higher MBI scores (rt-PA: 70.31 ± 7.05 at 3 months, 80.68 ± 8.68 at 6 months; urokinase: 64.84 ± 6.51 at 3 months, 71.65 ± 7.20 at 6 months) and higher GOS scores compared with the acute and subacute subgroups at both follow-up time points (*p* < 0.05; Figure 4). No statistically significant differences were observed between the acute and subacute subgroups for either MBI or GOS scores.

#### Between-group comparisons (rt-PA vs. Urokinase)

**Figure 5:**
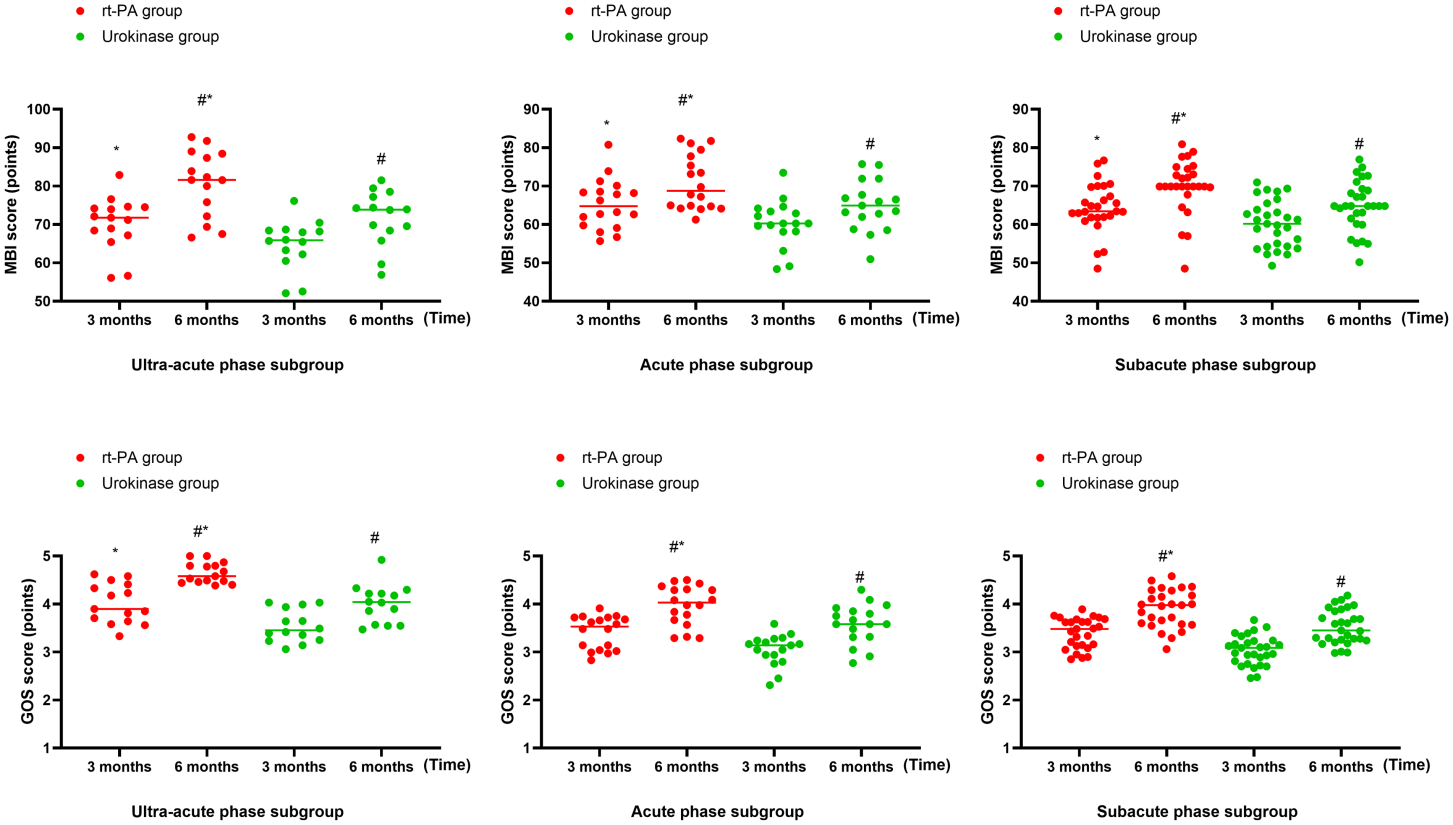
t-PA versus urokinase comparison of functional outcomes (MBI and GOS) within each time-window subgroup. Same outcome measures and time points as in Figure 4. #*p* < 0.05 vs. 3 months; *p < 0.05 vs. urokinase group (Bonferroni-adjusted). Independent-samples *t*-test was used for MBI; Mann–Whitney *U* test was used for GOS.

When comparing the two treatment arms within each time-window subgroup, the rt-PA group demonstrated significantly higher MBI and GOS scores at both 3 and 6 months postoperatively across all three subgroups (all Bonferroni-adjusted *p* < 0.017; Figure 5). In the ultra-acute phase subgroup, MBI scores at 6 months were 80.68 ± 8.68 in the rt-PA group versus 71.65 ± 7.20 in the urokinase group (mean difference 9.03 points, 95% CI: 3.07–14.99; Cohen’s *d* = 1.13), and GOS scores at 6 months were 4.67 ± 0.49 versus 4.07 ± 0.73, respectively. Similar patterns of rt-PA superiority were observed in the acute and subacute phase subgroups (Figure 5). These results indicate that rt-PA was associated with better functional independence and overall prognosis at medium-term follow-up than urokinase across all treatment time windows.

### Distribution of TAFI gene subtypes

The distribution of the three TAFI Thr325Ile genotypes (Thr325 [CC], Thr325Ile [CT], and Ile325 [TT]) and allele frequencies across subgroups is presented in Tables 3, 4. Notably, the genotype distribution in all subgroups of both treatment arms conformed to Hardy–Weinberg equilibrium (all *p* > 0.05), confirming that the study sample was representative of the population from which it was drawn.

**Table 3:** Proportion of the three TAFI gene subtypes in the three subgroups of the rt-PA group and urokinase group.

*rt-PA group*							
Subgroups	Cases	Thr325 (CC)	Thr325Ile (CT)	Ile325 (TT)	T allele	C allele	HWE (P)
Ultra-acute	15	1 (6.67)	2 (13.33)	12 (80.00)	26 (86.67)	4 (13.33)	0.654
Acute	18	4 (22.22)	10 (55.56)	4 (22.22)	18 (50.00)	18 (50.00)	0.359
Subacute	27	7 (25.93)	12 (44.44)	8 (29.63)	28 (51.85)	26 (48.15)	0.341
*p*		0.009			0.036		

*Urokinase group*							
Subgroups	Cases	Thr325 (CC)	Thr325Ile (CT)	Ile325 (TT)	T allele	C allele	HWE (P)
Ultra-acute	14	2 (14.29)	8 (57.14)	4 (28.57)	16 (57.14)	12 (42.86)	0.646
Acute	17	3 (17.65)	10 (58.82)	4 (23.53)	18 (52.94)	16 (47.06)	0.305
Subacute	29	7 (24.14)	12 (41.38)	10 (34.48)	32 (55.17)	26 (44.83)	0.099
*p*		0.945			0.744		

Allele frequencies are calculated from total alleles (2n). Values are presented as *n* (%). HWE, Hardy–Weinberg equilibrium goodness-of-fit *p*-value. Within the rt-PA group, the between-subgroup genotype difference corresponds to an odds ratio for Ile325 (TT) carriage in the ultra-acute versus combined acute + subacute subgroups of 10.46 (95% CI: 2.51–43.67). No corresponding signal was observed within the urokinase group.

**Table 4:** Proportion of the three TAFI gene subtypes between the rt-PA group and urokinase group in the three subgroups.

Subgroup	Group	Cases	Thr325 (CC)	Thr325Ile (CT)	Ile325 (TT)	T allele	C allele	OR for TT (95% CI)
Ultra-acute	rt-PA	15	1 (6.67)	2 (13.33)	12 (80.00)	26 (86.67)	4 (13.33)	10.00 (1.80–55.71)
	Urokinase	14	2 (14.29)	8 (57.14)	4 (28.57)	16 (57.14)	12 (42.86)	Reference
	*p*		0.012			0.020		
Acute	rt-PA	18	4 (22.22)	10 (55.56)	4 (22.22)	18 (50.00)	18 (50.00)	0.93 (0.20–4.31)
	Urokinase	17	3 (17.65)	10 (58.82)	4 (23.53)	18 (52.94)	16 (47.06)	Reference
	*p*		>0.999			>0.999		
Subacute	rt-PA	27	7 (25.93)	12 (44.44)	8 (29.63)	28 (51.85)	26 (48.15)	0.80 (0.26–2.43)
	Urokinase	29	7 (24.14)	12 (41.38)	10 (34.48)	32 (55.17)	26 (44.83)	Reference
	*p*		0.943			0.783		

Allele frequencies are calculated from total alleles (2n). Values are presented as *n* (%). Odds ratios (ORs) with 95% confidence intervals refer to carriage of the Ile325 (TT) genotype in the rt-PA group versus the urokinase group within each time-window subgroup.

Within the rt-PA group (Table 3), a statistically significant difference in genotype distribution was observed among the three time-window subgroups (*p* = 0.009). The ultra-acute phase subgroup exhibited a markedly higher frequency of the Ile325 (TT) genotype (80.00%) compared with the acute (22.22%) and subacute (29.63%) subgroups. Correspondingly, the T allele frequency was higher in the ultra-acute subgroup (86.67%) compared with the acute (50.00%) and subacute (51.85%) subgroups (*p* = 0.036), with a reciprocally lower C allele frequency (13.33% vs. 50.00 and 48.15%). By contrast, no significant differences in TAFI genotype or allele distribution were observed among the three subgroups of the urokinase group (*p* = 0.945 for genotype; *p* = 0.744 for allele frequency; Table 3).

Between-group comparison within each time-window subgroup (Table 4) revealed that in the ultra-acute phase, the rt-PA group had a significantly higher frequency of the Ile325 genotype (80.00% vs. 28.57%; odds ratio for TT carriage 10.00; 95% CI: 1.80–55.71; *p* = 0.012), higher T allele frequency (86.67% vs. 57.14%; *p* = 0.020), and lower Thr325 genotype and C allele frequencies compared with the urokinase group. In the acute and subacute phase subgroups, no significant between-group differences in genotype or allele distribution were detected (all *p* > 0.05; Table 4).

### Hematoma clearance rate, perihematomal edema volume, and relative edema volume

#### Within-group comparisons across time-window subgroups

**Figure 6:**
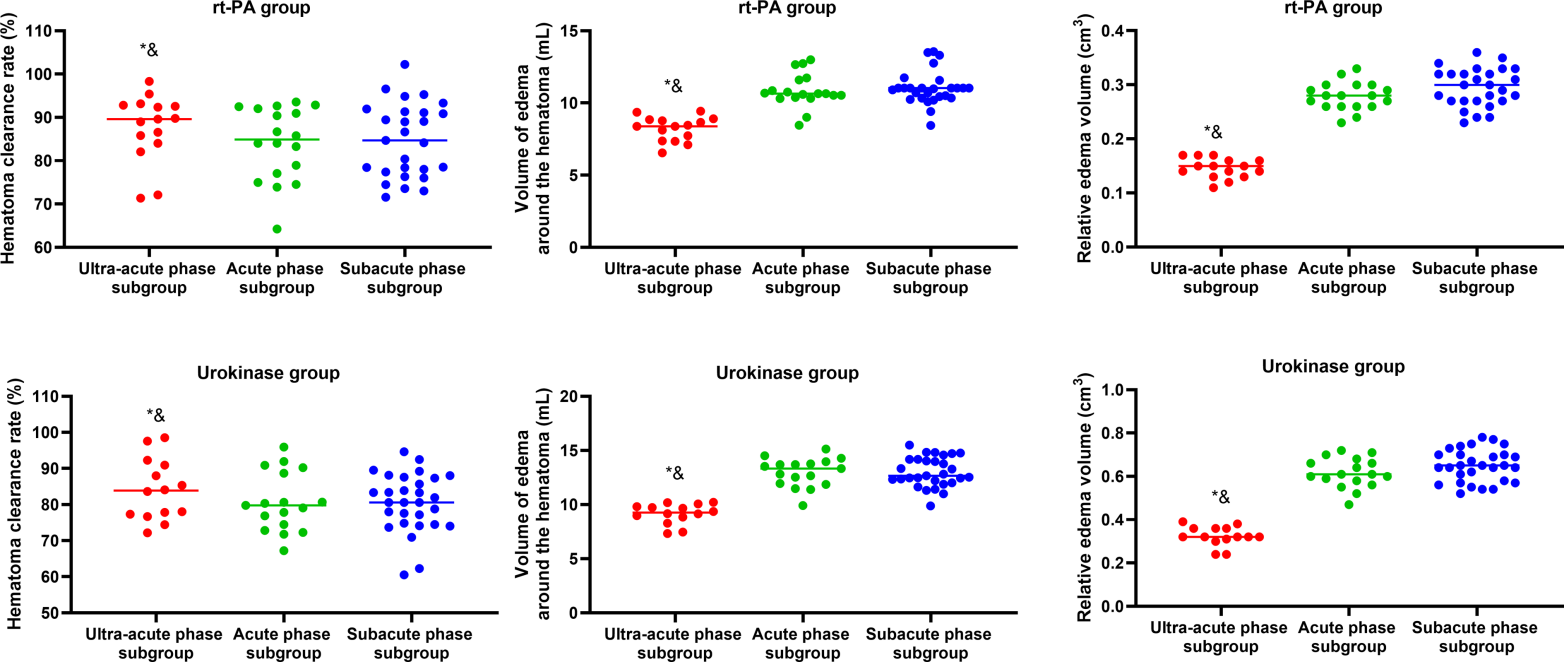
Radiological outcomes across time-window subgroups within each treatment group. *p < 0.05 vs. acute subgroup; ^&^*p* < 0.05 vs. subacute subgroup. Sample sizes as in Figure 2. One-way ANOVA was used for between-subgroup comparisons. Conclusions remained unchanged after ANCOVA adjustment for baseline hematoma volume and location.

Hematoma clearance rate, absolute perihematomal edema volume, and relative edema volume all varied significantly by treatment time window in both treatment groups (Figure 6). In the rt-PA group, the ultra-acute phase subgroup achieved a higher hematoma clearance rate (87.65 ± 7.75%) compared with the acute (84.01 ± 8.46%) and subacute (84.68 ± 8.53%) subgroups. The ultra-acute subgroup also demonstrated a smaller absolute perihematomal edema volume (8.22 ± 0.84 mL vs. 10.85 ± 1.16 mL and 11.03 ± 1.15 mL, respectively) and a smaller relative edema volume (0.14 ± 0.01 vs. 0.27 ± 0.02 and 0.29 ± 0.03, respectively; all *p* < 0.05). A similar pattern was observed in the urokinase group, where the ultra-acute subgroup showed favorable values compared to the later time-window subgroups (Figure 6).

To account for potential confounding by baseline hematoma characteristics, these comparisons were further analyzed using ANCOVA with baseline hematoma volume and location (lobar vs. deep) as covariates. After covariate adjustment, the statistical conclusions remained unchanged, confirming that the favorability of the ultra-acute treatment window was independent of baseline hematoma characteristics.

#### Between-group comparisons (rt-PA vs. Urokinase)

**Figure 7:**
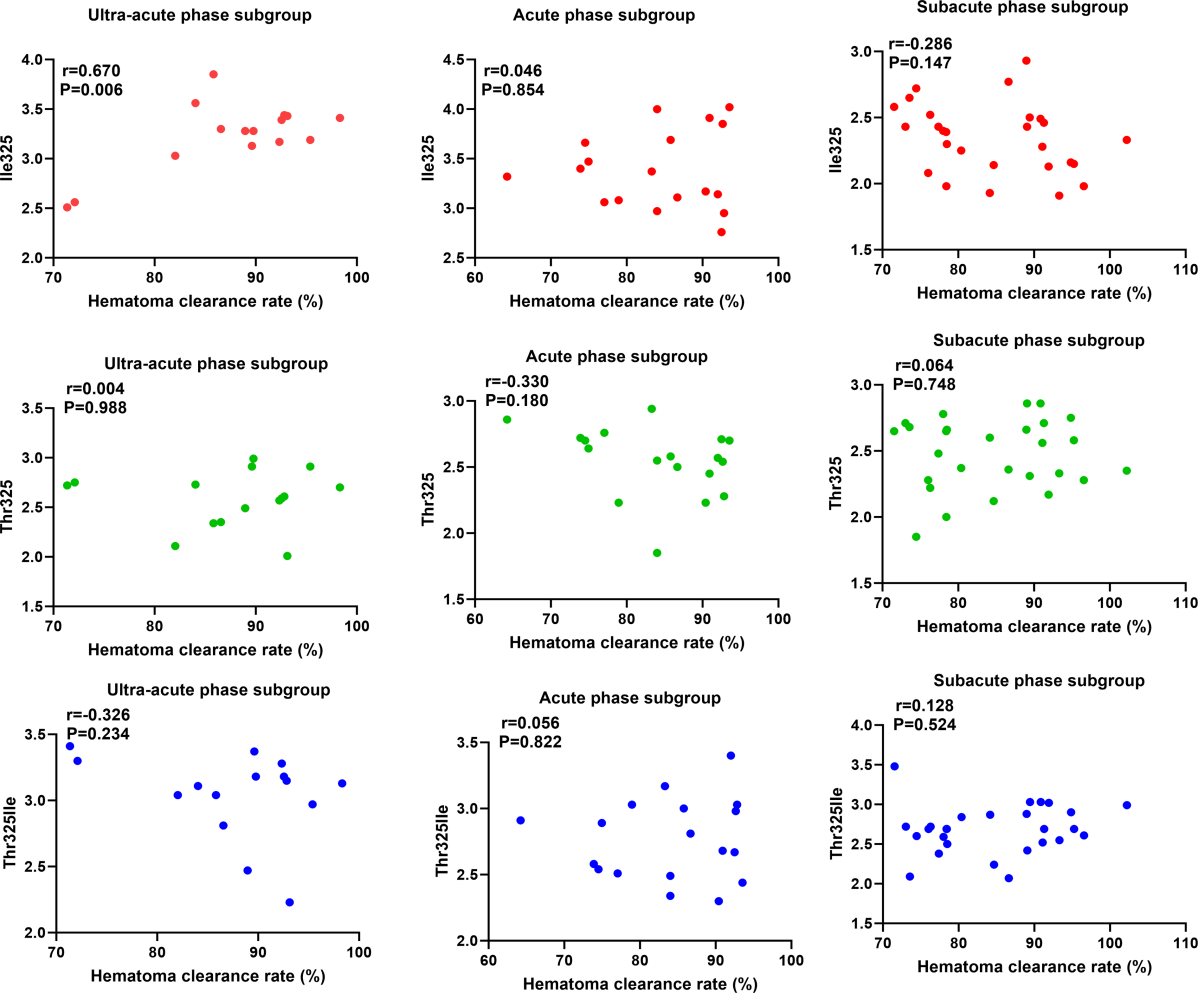
PA versus urokinase comparison of radiological outcomes within each time-window subgroup. *p < 0.05 vs. urokinase group (Bonferroni-adjusted). Sample sizes as in Figure 2. Independent-samples *t*-test was used; results remained significant after ANCOVA adjustment.

Direct comparison between treatment arms within each time-window subgroup demonstrated that the rt-PA group achieved significantly higher hematoma clearance rates, smaller perihematomal edema volumes, and smaller relative edema volumes than the urokinase group across all three time windows (all Bonferroni-adjusted *p* < 0.017; Figure 7). In the ultra-acute phase subgroup, the hematoma clearance rate was 87.65 ± 7.75% in the rt-PA group versus 84.05 ± 8.46% in the urokinase group (mean difference 3.60 percentage points, 95% CI: −2.62 to 9.82; interpretation should account for this CI), the perihematomal edema volume was 8.22 ± 0.84 mL versus 9.15 ± 0.92 mL (mean difference 0.93 mL, 95% CI: 0.27–1.59), and the relative edema volume was 0.14 ± 0.01 versus 0.32 ± 0.04, respectively. The relative edema volume difference was particularly pronounced, with the urokinase group displaying values approximately twice those of the rt-PA group across all subgroups. These differences remained statistically significant after adjustment for baseline hematoma volume and location using ANCOVA (Figure 7).

### Correlation between TAFI gene subtypes and hematoma clearance rate in the rt-PA Group

**Figure 8:**
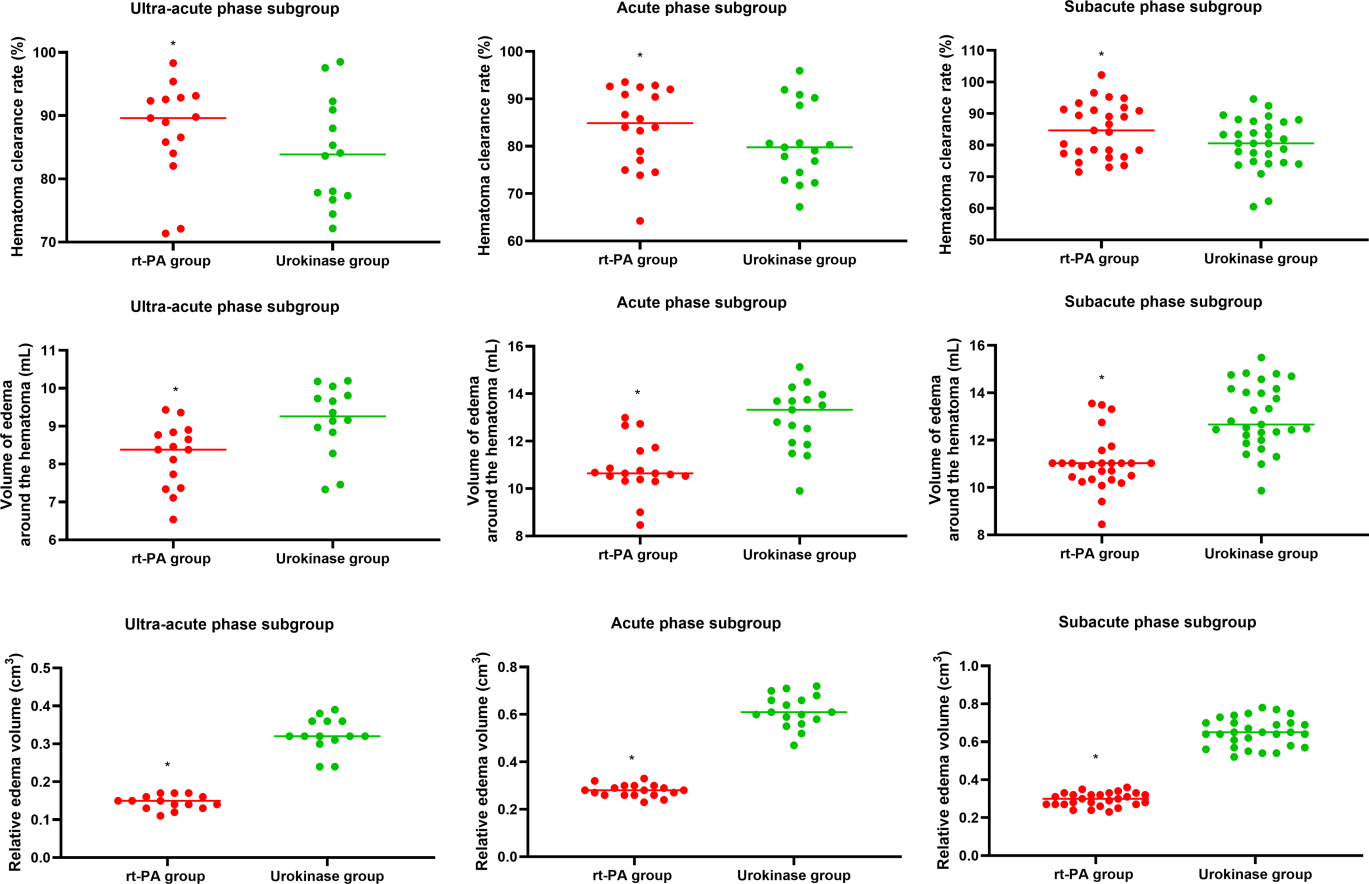
Correlation between TAFI Thr325Ile genotypes and hematoma clearance rate within the rt-PA group, by time-window subgroup. Scatter plots with Pearson correlation coefficients are shown for each genotype–subgroup combination. A significant positive correlation was observed between the Ile325 (TT) genotype and hematoma clearance rate in the ultra-acute subgroup (*r* = 0.670; 95% CI: 0.247–0.880; *p* = 0.006). No significant correlations were observed for the Thr325 (CC) or Thr325Ile (CT) genotypes. TAFI, thrombin-activatable fibrinolysis inhibitor.

Pearson correlation analysis was performed to explore the relationship between the three TAFI genotypes and hematoma clearance rate within each time-window subgroup of the rt-PA group (Figure 8). A statistically significant positive correlation was identified between the Ile325 (TT) genotype and hematoma clearance rate in the ultra-acute phase subgroup (*r* = 0.670, 95% CI: 0.247–0.880; *p* = 0.006), suggesting that patients carrying the Ile325 genotype who were treated with rt-PA within the ultra-acute window experienced more effective hematoma dissolution. In contrast, neither the Thr325 (CC) genotype nor the Thr325Ile (CT) genotype demonstrated a statistically significant correlation with hematoma clearance rate in any of the three time-window subgroups (all *p* > 0.05; Figure 8). These findings are consistent with a genotype-specific, time-dependent association between the TAFI Ile325 variant and rt-PA–mediated fibrinolysis, although this observation requires validation in adequately powered prospective studies.

### Exploratory subgroup analyses by hemisphere and hematoma volume stratum

Two additional exploratory subgroup analyses were performed to examine the robustness of the principal between-group findings. When patients were stratified by dominant-hemisphere involvement (left vs. right hemisphere), the direction and statistical significance of the rt-PA versus urokinase comparison were preserved within both strata for the principal neurological and functional endpoints (Supplementary Table S1). As anticipated from established prognostic literature, patients with dominant- (left-) hemisphere hemorrhage demonstrated somewhat poorer functional scores at 3 and 6 months than patients with non-dominant-hemisphere involvement within both treatment arms; however, the relative advantage of rt-PA over urokinase was consistent across hemisphere strata. When patients were stratified by baseline hematoma volume (30–50 mL vs. 51–80 mL) according to the threshold underpinning the volume component of the ICH Score (23, 24), outcomes were also more favorable in the smaller-volume stratum within both treatment arms, but the rt-PA versus urokinase difference persisted in each stratum (Supplementary Table S2). These exploratory analyses suggest that the principal between-treatment finding is not driven by imbalances in hemisphere laterality or baseline hematoma volume; however, the small per-stratum sample sizes preclude firm conclusions, and these results should be regarded as hypothesis-generating.

## Discussion

Minimally invasive thrombolytic therapy is an evolving strategy for the management of spontaneous ICH (25). CT-guided injection of thrombolytic agents through puncture channels can liquefy and help evacuate hematomas with acceptable safety (26), and minimally invasive approaches, including endoscopic surgery and stereotactic aspiration, have been associated with improved long-term outcomes relative to traditional craniotomy in selected patients, particularly those with deep hemorrhages (27). The MISTIE III trial established the technical feasibility and safety of image-guided catheter drainage with alteplase, though the primary functional outcome was not met at the cohort level (9). Subsequent secondary analyses have emphasized the importance of achieving a hematoma volume threshold (≤15 mL residual) and have re-examined the data using Bayesian methods, suggesting a non-trivial probability of benefit in patients reaching this target (25). More recently, the ENRICH trial demonstrated functional benefit for minimally invasive parafascicular surgery in lobar hemorrhages, further supporting the broader minimally invasive paradigm (10). In China, urokinase is widely used within this paradigm; however, its application in ICH thrombolysis lacks robust international validation (28). By contrast, rt-PA, the globally established thrombolytic for ischemic stroke, is being actively investigated for minimally invasive ICH evacuation (29). Our study compared these two agents within this therapeutic framework using a retrospective comparative cohort design.

Our findings suggest that rt-PA, administered via minimally invasive puncture, was associated with more favorable outcomes than urokinase across multiple domains. Patients in the rt-PA group demonstrated better neurological recovery on the NIHSS, mRS, and GCS, greater functional independence on the MBI, and more favorable prognosis on the GOS at discharge and at follow-up. The rt-PA group also achieved higher hematoma clearance rates and had less perihematomal edema. These differences remained statistically significant after Bonferroni adjustment for the three time-window between-group comparisons, and after adjustment for baseline hematoma volume and location using ANCOVA (Figures 3, 5, 7). However, this was a retrospective, non-randomized comparison; treatment allocation was driven in part by drug availability, physician familiarity, and patient/family preference, and unmeasured confounders, including factors that influenced the treating physician’s choice of agent, may have contributed to the observed differences. We therefore frame the findings as associations rather than as definitive evidence of therapeutic superiority.

The results also highlight the importance of treatment timing. Regardless of the thrombolytic agent used, patients treated within the ultra-acute window (3–6 h post-onset) showed more favorable outcomes than those treated in the acute (6–24 h) or subacute (24–72 h) phases (Figures 2, 4, 6). These outcomes included higher hematoma clearance, reduced edema, and improved neurological and functional scores. This is consistent with the established principle in stroke care that earlier intervention generally yields better results (30, 31). The absence of a significant difference between the acute and subacute subgroups further suggests that the greatest apparent benefit is concentrated within the first 6 h.

Beyond comparing agents and timing, we explored a potential genetic correlate of treatment response. TAFI is a key regulator of fibrinolysis and an endogenous inhibitor of plasmin-mediated clot lysis, and rt-PA acts upstream of this pathway by activating plasminogen (32). Previous research has linked TAFI levels and genetic polymorphisms to cardiovascular and thromboembolic risk (33), and the Schneider et al. functional study demonstrated that the Thr325 and Ile325 variants differ substantially with respect to thermal stability and antifibrinolytic activity (34). The clinical implications of TAFI polymorphisms in ICH have not been established, and prior pharmacogenomic work in stroke thrombolysis has predominantly focused on ischemic stroke and on other fibrinolytic regulators such as PAI-1 (35). In our cohort, the Ile325 genotype was more frequent in the ultra-acute subgroup of the rt-PA group than in later time windows and than in the corresponding urokinase subgroup (Tables 3, 4), and within the ultra-acute rt-PA subgroup the Ile325 genotype was positively correlated with hematoma clearance rate (*r* = 0.670, *p* = 0.006; Figure 8). One biologically plausible explanation is that the Ile325 variant, with its altered thermal stability and antifibrinolytic activity (34), modifies the balance between fibrinolysis and its inhibition during the early hours after hemorrhage when the clot is most amenable to thrombolytic dissolution. However, this remains a hypothesis. The present data are observational, the subgroup sizes are small, the genotype distribution itself differed between the rt-PA and urokinase ultra-acute strata in ways consistent with chance imbalance in a small non-randomized cohort, and we did not measure circulating TAFI antigen, TAFI activity, or downstream fibrinolytic markers that would be required to substantiate a mechanistic claim. Accordingly, the genotype association observed here should be considered hypothesis-generating rather than mechanistic.

### Clinical implications and future perspectives

If validated in adequately powered prospective studies, the association of the TAFI Ile325 genotype with enhanced rt-PA performance in the ultra-acute phase would raise the possibility of genotype-guided treatment selection. Translating such a finding would require rapid, point-of-care genotyping assays capable of delivering results within the narrow therapeutic window, characterization of the Ile325 prevalence in relevant populations, and an assessment of the feasibility and cost-effectiveness of rapid TAFI genotyping in the emergency setting. Equitable access to biomarker-guided therapy is an additional consideration that warrants attention in future study design. Our study does not provide sufficient evidence to recommend routine genotyping; rather, it generates a hypothesis that requires validation in larger, prospective, multicenter studies.

### Limitations

Several limitations should be considered when interpreting these findings. First, and most importantly, the single-center, retrospective, non-randomized design carries a substantial risk of selection bias and confounding by indication. Treatment allocation in routine practice was driven by drug availability, prescriber preference, patient/family financial considerations, and patient/family choice after counseling; consequently, the rt-PA and urokinase groups, although well balanced on the measured baseline variables presented in Table 1, may differ on unmeasured factors (e.g., socioeconomic status, time-of-day-related staffing patterns, evolving institutional preferences during the two-year study window). Although we adjusted for baseline hematoma volume and location using ANCOVA and performed exploratory subgroup analyses by hemisphere and volume stratum, these adjustments cannot fully eliminate residual confounding, and the principal between-treatment association should be interpreted accordingly.

Second, the subgroup sample sizes, particularly within each treatment-by-time-window stratum and within genotype strata, are modest, limiting statistical power for the subgroup and genotype interaction analyses. The associations involving treatment time windows and TAFI genotypes must therefore be considered preliminary and require replication in larger cohorts.

Third, the exploratory nature of the subgroup and genotype analyses, conducted without formal correction for multiple comparisons (apart from Bonferroni adjustment of the three primary time-window between-group contrasts), increases the risk of Type I error. Given the number of stratified comparisons performed across time windows, TAFI genotypes, hemisphere laterality, and hematoma-volume strata, some of the unadjusted significant findings may reflect chance associations rather than true effects, and Type II error is also possible in the smallest strata. Confirmatory studies should pre-specify a multiplicity-adjusted analysis plan.

Fourth, the use of urokinase as a comparator, while reflective of local clinical practice, limits the generalizability and international comparability of the findings; future studies should consider placebo-controlled designs or internationally recognized active comparators such as alteplase or tenecteplase.

Fifth, we examined only the Thr325Ile polymorphism of TAFI. The complex process of fibrinolysis involves numerous other genes and regulators (e.g., PAI-1, fibrinogen, plasminogen), and their potential interactions were not assessed. In addition, circulating TAFI antigen and activity levels and downstream fibrinolytic markers were not measured, precluding direct mechanistic inference.

Finally, although interobserver reliability for the radiological measurements was excellent in our subset analysis (Supplementary Table S3), the volumetric assessments were performed at a single center using a single imaging platform, which may limit transportability of the imaging findings to other settings.

## Conclusion

In this retrospective comparative study, minimally invasive hematoma evacuation with rt-PA was associated with more favorable neurological, functional, and radiological outcomes than treatment with urokinase in patients with spontaneous supratentorial ICH. The data further suggest that the ultra-acute phase (within 3–6 h of onset) represents the most favorable treatment window. The TAFI Ile325 genotype warrants further investigation as a candidate pharmacogenomic marker for enhanced rt-PA response during this early period. Given the retrospective, non-randomized design and the exploratory nature of the subgroup analyses, these findings are hypothesis-generating and require validation in larger, multicenter, prospective studies before any change in clinical practice can be considered.

## Data Availability

The raw data supporting the conclusions of this article will be made available by the authors, without undue reservation.
