# Supplementary material for: Efficacy of rt-PA versus Urokinase in early time windows for minimally invasive intracerebral hemorrhage evacuation and exploratory TAFI pharmacogenomics
Source: Front Neurol. 2026 Aug 3;17:1830505. doi: 10.3389/fneur.2026.1830505 (PMC13477919; doi:10.3389/fneur.2026.1830505)
Supplement: Supplementary file 1 [file Table_1.DOCX]

**Supplementary Table S1. Key outcomes stratified by dominant hemisphere status within each treatment group (exploratory subgroup analysis).**

| Stratum | Group | n | NIHSS at 3 mo (mean ± SD) | mRS at 3 mo (mean ± SD) | MBI at 6 mo (mean ± SD) | GOS at 6 mo (mean ± SD) | Hematoma clearance (%) | rt-PA vs. UK P (NIHSS / MBI) |
| --- | --- | --- | --- | --- | --- | --- | --- | --- |
| Left (dominant) | rt-PA | 35 | 13.85 ± 1.62 | 3.05 ± 0.45 | 74.55 ± 7.82 | 4.25 ± 0.50 | 84.95 ± 8.25 | <0.001 / <0.001 |
|  | Urokinase | 33 | 19.05 ± 1.95 | 3.85 ± 0.52 | 65.42 ± 6.85 | 3.65 ± 0.68 | 80.85 ± 8.42 |  |
| Right (non-dominant) | rt-PA | 25 | 12.15 ± 1.45 | 2.80 ± 0.42 | 79.65 ± 7.95 | 4.45 ± 0.55 | 86.12 ± 8.36 | <0.001 / <0.001 |
|  | Urokinase | 27 | 17.65 ± 1.78 | 3.55 ± 0.48 | 70.32 ± 7.18 | 3.85 ± 0.72 | 81.65 ± 8.38 |  |

*Note. Left hemisphere was designated as dominant for this predominantly right-handed cohort. Direction and statistical significance of the rt-PA versus urokinase comparison are preserved within both hemisphere strata. P values were derived from independent-samples t-tests for NIHSS and MBI; comparable directional findings were observed for mRS, GOS, and hematoma clearance (Mann–Whitney U for ordinal scales). Mean difference in 3-month NIHSS (rt-PA vs. urokinase) within the dominant-hemisphere stratum = 5.20 (95% CI: 4.32–6.08); within the non-dominant stratum = 5.50 (95% CI: 4.43–6.57). NIHSS = National Institutes of Health Stroke Scale; mRS = modified Rankin Scale; MBI = Modified Barthel Index; GOS = Glasgow Outcome Scale.*

**Supplementary Table S2. Key outcomes stratified by baseline hematoma volume (30–50 mL vs. 51–80 mL) within each treatment group (exploratory subgroup analysis).**

| Stratum | Group | n | NIHSS at 3 mo (mean ± SD) | mRS at 3 mo (mean ± SD) | MBI at 6 mo (mean ± SD) | GOS at 6 mo (mean ± SD) | Hematoma clearance (%) | rt-PA vs. UK P (NIHSS / MBI) |
| --- | --- | --- | --- | --- | --- | --- | --- | --- |
| 30–50 mL | rt-PA | 35 | 12.15 ± 1.42 | 2.75 ± 0.45 | 78.85 ± 8.12 | 4.45 ± 0.55 | 86.85 ± 8.32 | <0.001 / <0.001 |
|  | Urokinase | 33 | 17.35 ± 1.85 | 3.45 ± 0.55 | 70.45 ± 7.32 | 3.95 ± 0.70 | 82.15 ± 8.42 |  |
| 51–80 mL | rt-PA | 25 | 14.45 ± 1.62 | 3.20 ± 0.48 | 73.25 ± 7.78 | 4.10 ± 0.52 | 83.95 ± 8.46 | <0.001 / <0.001 |
|  | Urokinase | 27 | 19.85 ± 2.05 | 3.95 ± 0.60 | 64.85 ± 6.78 | 3.55 ± 0.65 | 79.85 ± 8.38 |  |

*Note. Mean difference in 3-month NIHSS (rt-PA vs. urokinase) within the 30–50 mL stratum = 5.20 (95% CI: 4.40–6.00); within the 51–80 mL stratum = 5.40 (95% CI: 4.36–6.44). NIHSS = National Institutes of Health Stroke Scale; mRS = modified Rankin Scale; MBI = Modified Barthel Index; GOS = Glasgow Outcome Scale.*

**Supplementary Table S3. Interobserver reliability of volumetric CT measurements.**

| Measurement | Number of paired re-measurements | ICC(2,1) | 95% CI | Mean absolute between-reader difference |
| --- | --- | --- | --- | --- |
| Hematoma volume | 30 (25% of total cohort) | 0.94 | 0.88–0.97 | 0.7 mL |
| Perihematomal edema volume | 30 (25% of total cohort) | 0.91 | 0.82–0.95 | 1.1 mL |
| Combined (hematoma + edema) region | 30 (25% of total cohort) | 0.93 | 0.85–0.96 | 1.5 mL |

*Note. ICC(2,1) = two-way random-effects, single-rater, absolute-agreement intraclass correlation coefficient. All measurements were performed on the IntelliSpace Portal Philips Nebula workstation by two experienced neuroradiologists who were independently blinded to treatment allocation, clinical outcomes, and each other’s readings.*
